# Effects of Adding Sodium Nitroprusside to Semen Diluents on Motility, Viability and Lipid Peroxidation of Sperm in Holstein Bulls

**DOI:** 10.22074/ijfs.2015.4611

**Published:** 2015-12-23

**Authors:** Hamidreza Khodaei, Mohammad Chamani, Behnaz Mahdavi, Ali Asghar Akhondi

**Affiliations:** 1 Depatment of Animal Science, Golpayegan Branch, Islamic Azad University, Golpayegan, Iran; 2 Department of Animal Physiology, Faculty of Agriculture and Natural Resources, Science and Research Branch, Islamic Azad University, Tehran, Iran

**Keywords:** Sperm Motility, Nitric Oxide, Lipid Peroxidation, Spermatozoa

## Abstract

**Background:**

Nitric oxide (NO) that plays important role in all sexual activities of animals is made from the amino acid L-arginine by the enzymatic action of NO synthase (NOS). NO makes a band with sulfur-iron complexes, but due to production of steroid sexual hormones related to the enzymes involved in this complex, NO can change the activity of these enzymes. NO affects many cells including vein endothelial cells, macrophages and mast cells. These cells are also found in Leydig cells; therefore, they are important source of NO in testis tissue. Therefore, minimizing damages to sperm at the time of freezing thawing process are really important. The aim of this study was to determine the appropriate NO concentration to be added to the freezing extender to improve the quality of thawed sperm.

**Materials and Methods:**

In this experimental randomized study, sperms of four Holstein bulls with an average age of 4 were collected twice a week for 3 weeks. They received sodium nitroprusside (SNP) in concentrations of 0, 10, 50 and 100 nmol/ml. Data analysis was performed using the special issue and static (SAS) 98 software. Also, mean comparison was done using Duncan’s multiple ranges test (P<0.05).This research was conducted at the laboratory of Science and Research Branch, Islamic Azad University, Tehran at spring and summer of 2013.

**Results:**

All concentrations of SNP used was found to increase motility and viability of spermatozoa at 1, 2 and 3 hours after thawing, significantly (P<0.05), but there was no significant difference at zero time. Different concentrations of SNP reduced the membrane lipid peroxidation level of sperm and increased acrosome membranes integrity, implying that SNP generally improved samples membranes, especially in 50 and 100 nmol/ml concentrations.

**Conclusion:**

According to the obtained results, addition of SNP to semen diluents increases motility and viability of spermatozoa. Also, it reduces membrane lipid peroxidation level that leads to improved sperm function.

## Introduction

Fertility is very important in livestock, especially in bulls, because obtained sperm are used in insemination of other cows. Freezing thawing process causes sperm damage, like loss of lipid membrane integrity, mitochondria activity, and acrosome membrane integrity, leading to a reduction in motility, viability and fertility of sperm (1).

Therefore, the most important factor in freezing thawing process is to minimize the sperm damages. Freezing-thawing process causes physiochemical stresses on sperm membrane, leading to decrease quality, motility, viability and fertility of sperm (2). Two types of damages are as follows: Production of lots of free radicals and occurrence of peroxidation of phospholipids in sperm membrane which increases the level of fatty acid oxidation including malondiealdehyde (MDA) (3-5). Nitric oxide (NO) as an active non organic molecule is spreadable and free and non stable which is considered as endothelium-derived relaxing factor in veins. It is made in body from the amino acid L-arginine by the enzymatic action of NO synthase (NOS). After making band with sulfur-iron complexes NO changes the activity of these enzymes. NO is an important transmitter molecule in mammalian cells including human and plays a main role in physiological and pathological processes (6, 7). Effect of NO has been observed on many physiological activities of organs, especially male sexual system, like sperm motility, acrosomic reaction, chemotaxis, ability of sperm to bind to the egg, spermatogenesis and balancing the action of hypothalamic-pituitary-gonadal axis (8). 

There are few studies about the effects of NO on quality of bulls’ sperm, especially on measurement of membrane damage via measuring MDA level. The aim of this study was to determine the appropriate NO concentration to be added to the freezing extender in order to improve the quality of thawed sperm.

## Materials and Methods

This research was conducted at the laboratory of Science and Research Branch, Islamic Azad University, Tehran at spring and summer of 2013. In this experimental randomized study, four Holstein bulls with a mean age of four years and appropriate quantitative and qualitative characteristics of sperm were selected one month before the test. Bulls (jahed research center) were kept in separate boxes and fed according to the National Research Council (NRC, 2000) (9).

### Sperm method

Sperm was collected twice a week for 3 weeks. Then samples were transferred to laboratory rapidly and incubated at 37˚C in a bain-marie. After semen volume, population and sperm motility of each bull were determined, samples were mixed, divided in four aliquots, and diluted using Bioxell (Bioxell Inc., USA) containing various concentrations of sodium nitroprusside (SNP). So, four SNP treatments including 0 (control), 10, 50 and 100 nmol/ml were studied for 6 weeks (replications). After dilution and packing in 0.5 cc pivots, 2 pivots of each sample were transferred to laboratory, and quality and quantity of sperms were analyzed using computer-aided system analysis (CASA). Samples were then frozen using digital freezer and kept in nitrogen tanks. At least after 24 hours, thawed samples were incubated and motility percentage, viability, acrosome status and MDA level were analyzed at 0, 1, 2, and 3 hours being kept at 37˚C. Freezing or cry therapy is a process of longterm preservation of cells and tissues at very low temperatures. To perform the insemination, the frozen sperms should reach the proper temperature. Payout melting process was performed in a water bath of 32-35˚C.

### Measuring the motility of spermatozoa

Samples were placed in warm water bath at 37˚C for 3 minutes, 5 μl was transferred on a slide, and covered with a covers lip of 18×18 mm. Sperm analysis was done using CASA at 0, 1, 2 and 3 hours after thawing.

### Measuring viability percentage of sperm

This was carried out using eosin staining method, while nigrosin was used here as background color.

### Measuring the level of membrane lipid peroxidation

MDA level was measured by Esterbauer and Cheeseman method (1990) using a spectrophotometer (spectrophotometer uv-varian-CARY50 scan visible, Thermo, USA) at 532 nm wavelength. This method is carried out on the bases of reaction of MDA with thiobarbituric acid (TBA), leading to elimination of double water molecules.

### Measuring acrosome integrity of sperm

In fertility, determining the acrosome percentage is a morphological method for measuring viability of sperm after thawing. For this, sperm motility is inhibited initially by mixing semen with Glutaraldehyde 2% in phosphate buffer (NJ, USA). This buffer fixes the membrane and prohibits its deterioration. About 0.5 ml of semen sample was placed on a slide and was mixed with one drop of buffer. This mixture was scattered on slide, and slide was observed using a contrast phase microscope with ×1000 to ×3000 magnifications.

### Measuring sperm membrane functionality

About 250 ml of diluted semen was incubated in 1 ml of hypo-osmotic swelling test (HOST) solution with osmolality of 100 m Osm/kg for 40 to 60 minutes and analyzed around 400 spermatozoa using a contrast phase microscope with ×1000 to ×3000 magnifications.

### Statistical analysis

Data were analyzed using the special issue and static (SAS) 98 software. ANOVA was used to compare the mean values, while Duncan’s multiple range test was carried out to analyze the difference between control and treatment groups. The significance level was set at P<0.05.

## Results

### Total motility and progressive motility of spermatozoa

According to results, SNP increased total motility of sperms significantly (P<0.05) in comparison with control group (Table 1). It increased progressive motility in 1, 2 and 3 hours after thawing, but at zero hour (immediately after thawing), motility increased only by 100 nmol/ml, indicating that it is not significant (P>0.05). Increase in mobility by SNP was also not significant before freezing (P>0.05). Our findings showed that there is no significant difference in 100-nmol treatment regarding total motility of spermatozoa (P>0.05) (Table 1).

In case of progressive motility of spermatozoa, the first and second hours after thawing showed the best results, while among SNP concentrations, 100 nmol showed a significant difference compared to the control (Table 2).

**Table 1:** The effects of different concentration of SNP on total motility of spermatozoa at various thawing times


Treatment (SNP)	0 nmol/ml	10 nmol/ml	50 nmol/ml	100 nmol/ml

Before freezing	74.08 ± 1.08a	77.08 ± 1.80a	76.5 ± 1.08^a^	79.75 ± 1.85^a^
Immediately after thawing	57.00 ± 2.06^a^	50.16 ± 1.08^a^	49.25 ± 1.08^b^	56.08 ± 2.02^a^
1 hour after thawing	45.33 ± 1.09^a^	54.08 ± 2.8^b^	53.75 ± 2.8^b^	58.41 ± 2.10^b^
2 hours after thawing	41.42 ± 1.02^a^	48.33 ± 2.8^b^	46.83 ± 2.3^ab^	50.66 ± 2.10^b^
3 hours after thawing	33.83 ± 1.03^a^	41.66 ± 1.06^b^	39.58 ± 1.05^b^	45.08 ± 1.09^b^


The data are presented as mean ± SD. SNP; Sodium nitroprusside. Common letters in each row indicate no significant difference (P>0.05).

**Table 2:** The effects of different concentration of SNP on progressive motility of spermatozoa at various thawing times


Treatment (SNP)	0 nmol/ml	10 nmol/ml	50 nmol/ml	100 nmol/ml

Before freezing	39.75 ± 2.07^a^	42.41 ± 2.6^a^	45.91 ± 2.73^ab^	47.25 ± 2.8^b^
Immediately after thawing	37.08 ± 1.93^a^	34.25 ± 1.09^a^	34.75 ± 1.09^a^	40.08 ± 2.1^a^
1 hour after thawing	27.41 ± 1.07^a^	36.41 ± 1.7^b^	35.83 ± 1.7^b^	40.33 ± 2.5^b^
2 hours after thawing	24.66 ± 1.04^a^	29.91 ± 1.35^b^	30.08 ± 1.35^b^	35.33 ± 1.49^c^
3 hours after thawing	20.91 ± 1.03^a^	26.08 ± 1.30^b^	27 ± 1.32^b^	27.58 ± 1.36^b^


The data are presented as mean ± SD. SNP; Sodium nitroprusside. Common letters in each row indicate no significant difference (P>0.05).

### Sperm viability

SNP affected sperm viability and improved this parameter 1 hour after thawing. All concentrations of SNP increased viability significantly (<0.05). This increase was observed in second hour only in 10 and 50 nmol/ml concentrations. Three hour after thawing, all concentrations increased spermatozoa viability significantly (P<0.05). However, none of the used treatments could increase viability in zero treatment (Table 3).

### Lipid peroxidation

Different treatments of SNP reduced this parameter significantly (P<0.05) and damaged sperm membrane at 1 and 2 hours after thawing, but in zero time only 50 nmol caused a significant (P<0.05) reduction (Table 4).

### Acrosome integrity of sperm

Variance analysis of data showed that before freezing, SNP increased this parameter significantly (P<0.05) only in 100 nmol/ml treatment. Also immediately after thawing, 100 nmol treatment increased acrosome integrity significantly (P<0.05). In first hour after thawing, 50- and 100- nmol treatments increased this parameter, while all concentrations at second and third hours increased this parameter compared to the control group. The highest ratio of healthy spermatozoa was observed in 100-nmol treatment (Table 5).

### Sperm membrane functionality

SNP at all different hours after thawing and even before freezing could reduce the membrane damage significantly (P<0.05) in all treatments, while 100 nmol/ml treatment demonstrated the best results during the first hour after thawing (Table 6).

**Table 3:** The effects of different concentration of SNP on viability of spermatozoa at various thawing times


Treatment (SNP)	0 nmol/ml	10 nmol/ml	50 nmol/ml	100 nmol/ml

Before freezing	79.66 ± 1.07^a^	82.75 ± 1.5^a^	82.08 ± 1.09^a^	84.66 ± 1.72^a^
Immediately after thawing	65.00 ± 2.4^a^	59.50 ± 2.08^a^	59.08 ± 2.04^a^	64.58 ± 2.4^a^
1 hour after thawing	52.50 ± 1.07^a^	60.08 ± 1.09^b^	60.25 ± 1.6^b^	64.75 ± 1.77^b^
2 hours after thawing	48.91 ± 1.06^a^	55.33 ± 1.6^b^	54.33 ± 1.5^b^	57.33 ± 1.69^a^
3 hours after thawing	40.75 ± 1.03^a^	48 ± 1.38^ab^	46.5 ±1.3^b^	51.33 ± 2.01^c^


The data are presented as mean ± SD. SNP; Sodium nitroprusside. Common letters in each row indicate no significant difference (P>0.05).

**Table 4:** The effects of different concentration of SNP on level of membrane lipid peroxidation at various thawing times


Treatment (SNP)	0 nmol/ml	10 nmol/ml	50 nmol/ml	100 nmol/ml

Before freezing	0.20 ± 0.05^a^	0.19 ± 0.05^a^	0.15 ± 0.01^b^	0.16 ± 0.01^ab^
Immediately after thawing	0.17 ± 0.008^a^	0.15 ± 0.005^ab^	0.11 ± 0.001^b^	0.14 ± 0.005^c^
1 hour after thawing	0.21 ± 0.009^a^	0.14 ± 0.002^b^	0.14 ± 0.002^b^	0.12 ± 0.001^b^


The data are presented as mean ± SD. SNP; Sodium nitroprusside. Common letters in each row indicate no significant difference (P>0.05).

**Table 5:** The effects of different concentrations of SNP on acrosome integrity of spermatozoa at various thawing times


Treatment (SNP)	0 nmol/ml	10 nmol/ml	50 nmol/ml	100 nmol/ml

Before freezing	75. 5 ± 0.09^a^	74.16 ± 0.09^b^	72.83 ± 0.08^c^	78.66 ± 1.05^d^
Immediately after thawing	55.66 ± 0.01^a^	50.00 ± 0.09^b^	47.16 ± 0.08^c^	56.83 ± 0.02^d^
1 hour after thawing	38.33 ± 0.05^a^	38.16 ± 0.04^a^	39.33 ± 0.05^b^	46.33 ± 0.08^c^
2 hours after thawing	29.08 ± 0.04^a^	33.00 ± 0.06^b^	38.33 ± 0.08^c^	38.33 ± 0.08^c^
3 hours after thawing	20.25 ± 0.04^a^	22.16 ± 0.06^b^	25.16 ± 0.09^c^	27.00 ± 0.2^d^


The data are presented as mean ± SD. SNP; Sodium nitroprusside. Common letters in each row indicate no significant difference (P>0.05).

**Table 6:** The effects of different concentrations of SNP on membrane functionality at various thawing times


Treatment (SNP)	0 nmol/ml	10 nmol/ml	50 nmol/ml	100 nmol/ml

Before freezing	74.79 ± 0.06^a^	80.79 ± 0.42^b^	83.87 ± 0.5^c^	87.83 ± 0.61^d^
Immediately after thawing	68.54 ± 0.07^a^	76.45 ± 0.5^b^	79.54 ± 0.7^c^	82.25 ± 0.75^d^
1 hour after thawing	67.29 ± 0.05^a^	74.37 ± 0.3^b^	79.75 ± 0.5^c^	84.08 ± 0.57^d^
2 hours after thawing	69.37 ± 0.07^a^	75.00 ± 0.43^b^	79.37 ± 0.6^c^	83.66 ± 0.73^d^
3 hours after thawing	72.00 ± 0.07^a^	75.83 ± 0.18^b^	78.12 ± 0.3^c^	83.95 ± 0.57^d^


The data are presented as mean ± SD. SNP; Sodium nitroprusside. Common letters in each row indicate no significant difference (P>0.05).

## Discussion

NO is an important biological molecule which plays critical role in sperm physiology like sperm chemotaxis, sperm motility, and spermatogenesis (8). Several studies have reported sperm motility in low concentrations of NO in mice (10), sheep and human (11, 12). Direct and significant correlation was seen between NO concentrations and sedentary sperm numbers (13, 14). Some researchers have shown that NO is the main activator of guanylate cyclase (ubiquitous enzymes). It is noteworthy to mention that considerable amount of cyclic guanosine monophosphate (cGMP) obtained from this enzyme causes acrosome reaction, chemotaxis and sperm-egg reaction (8, 15). In this study, increasing effects of SNP on some parameters like total motility and progressive motility were in agreement with some studies (15-17), whereas some other studies have reported different results which is due to various SNP concentrations (11, 18-20). Previous studies have shown that concentrations higher than nmol per liter can reduce sperm activity due to toxicity of NO, but NO in low concentrations increases quality parameters of sperm. The concentrations of 50 and 100 nmol/ml are ideal which are reported by Sharma and Aqarwal (16). SNP increased sperm viability and improved sperm parameters which are consistent with some studies (16, 17) and not consistent with others (11, 19, 20). Membrane lipid peroxidation and acrosome integrity are considered as the best indications of a healthy sperm membrane. Our findings showed that different concentrations of SNP increased HOST and incubation times, while reduced MDA level in MDA test indicating controlling effect of NO on lipid per oxidation. Other studies also confirmed these results (17, 21). By studying lipid per oxidation process, we found that membrane injures resulted in reduction in motility and death of sperm. Disorders in balance of oxygen free radicals and in difference mechanism eliminating these radicals lead to reactive oxygen species (ROS) accumulation and induction of oxidative stress that results in damage of proteins, membrane lipids and other cell components (22). It has been reported that role of NO in prohibition of lipid per oxidation is associated with its ability in reaction with alkoxy lipid radicals (LX), lipid peroxyl (LOO) and chain-breaking oxidation (23, 24), which is in agreement with results of this study. Furthermore our results indicated that SNP improved the health of acrosome integrity compared to control, while the best effect was achieved for 100 nmol/ml treatment, which was in agreement with other studies (15). Researchers have emphasized mostly on morphological changes of sperm like twisted tail, injured membranes and injured acrosomes (25). Apparently, injured sperm in freezing process increases ROS production which causes damage to other normal sperms, but SNP reduces acrosome injury via decreasing oxidative stress.

## Conclusion

Based on the obtained results, SNP may improve motility, viability, acrosome and plasma membrane integrity during freezing-thawing process. Addition of SNP before freezing provides better results for bovine semen cryopreservation than its inclusion in the thawing extender.
